# Supplementary material for: Effect of TiO2 Nanoparticles and Extrusion Process on the Physicochemical Properties of Biodegradable and Active Cassava Starch Nanocomposites
Source: Polymers (Basel). 2023 Jan 20;15(3):535. doi: 10.3390/polym15030535 (PMC9918894; doi:10.3390/polym15030535)
Supplement: Supplementary file 1 [file polymers-15-00535-s001.zip › polymers-2074753-supplementary/polymers-2074753-supplementary.pdf]

polymers

MDPI

Supplementary Material

Effect of TiO_2 nanoparticles and extrusion process on the physicochemical properties of biodegradable and active cassava starch nanocomposites

Carolina Iacovone[1], Federico Yulita[1], Daniel Cerini[1], Daniel Peña[1], Roberto Candal[2], Silvia Goyanes[1,3], Lía I. Pietrasanta[3,4], Lucas Guz[2], Lucía Famá[1,3,*]

[1] Universidad de Buenos Aires, Facultad de Ciencias Exactas y Naturales, Departamento de Física. Laboratorio de Polímeros y Materiales Compuestos, Intendente Güiraldes 2160, C1428EGA Buenos Aires, Argentina.
[2] IIIA-UNSAM-CONICET, Instituto de Investigación e Ingeniería Ambiental, Escuela de Hábitat y Sostenibilidad, Campus Miguelete, 25 de mayo y Francia, 1650-San Martín, Provincia de Buenos Aires, Argentina
[3] CONICET - Universidad de Buenos Aires, Instituto de Física de Buenos Aires (IFIBA). Buenos Aires, Argentina.
[4] Universidad de Buenos Aires, Facultad de Ciencias Exactas y Naturales, Centro de Microscopías Avanzadas (CMA), Intendente Güiraldes 2160, C1428EGA Buenos Aires, Argentina.
* Correspondence lfama@df.uba.ar; Tel.: +54 11 5285 7511 (ext 57511)

Citation: To be added by editorial staff during production.

Academic Editor: Firstname Lastname

Received: date
Accepted: date
Published: date

Publisher's Note: MDPI stays neutral with regard to jurisdictional claims in published maps and institutional affiliations.

Copyright: © 2022 by the authors. Submitted for possible open access publication under the terms and conditions of the Creative Commons Attribution (CC BY) license (https://creativecommons.org/licenses/by/4.0/).

1. Antibacterial activity

Antibacterial effect was evaluated following the agar diffusion method in Muller-Hinton agar [1]. Briefly, Escherichia coli DH5α (E. coli) cultivated in LB medium at 37°C for 24 h were diluted to a final concentration of 108 CFU/mL. Then, 200 μL of the resulting bacterial suspension were evenly inoculated on agar plates containing Muller-Hinton agar. Circular samples of 2 cm diameter of each material were sterilized under UV radiation for 1 h and placed in the inoculated agar. Photographic records of the agar plates were taken after 24 h incubation at 37 °C.

Figure S1 shows the plates containing bacteria and the S80, S80-TiO2NP and S120-TiO2NP materials after 24 h incubation at 37°C. As can be seen in the picture, no inhibition halo was observed in any of the studied films. Similar results were reported for nanocomposites containing non diffusive antimicrobial nanoparticles in textiles [2] and in biodegradable polymers [3]. Other authors reported antimicrobial activity of TiO2 nanoparticles incorporated inside a polymer matrix [4–6], however, in these researches higher TiO2 concentrations were employed. Besides, in those works, the films were illuminated with UV light. In the presence of UV light, TiO2 generates highly oxidative reactive species that could kill bacteria or inhibit their growth [7]. Taking into consideration that the final disposition after usage of the films present in this study is composting, bacterial activity should not be examined under UV radiation, since the films would not be exposed to light. As a low concentration of TiO_2 was employed and samples were not illuminated under UV light, it was expected that the materials didn't present antimicrobial activity.

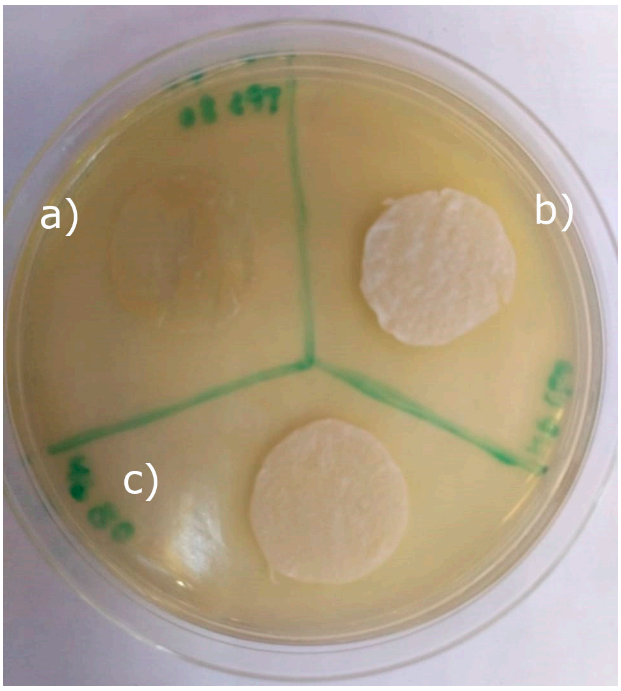

Figure S1: Antibacterial activity of (a) S_{120}, (b) S_{120}-TiO_2NP and (c) S_{80}-TiO_2NP against *E. coli* studied by the agar diffusion method.

References

1. Estevez-Areco, S.; Guz, L.; Candal, R.; Goyanes, S. Development of Insoluble PVA Electrospun Nanofibers Incorporating R-Limonene or β-Cyclodextrin/R-Limonene Inclusion Complexes. *J. Polym. Environ.* **2022**, 1–12, doi:https://doi.org/10.1007/s10924-022-02390-9.
2. Pinho, E.; Magalhães, L.; Henriques, M.; Oliveira, R. Antimicrobial Activity Assessment of Textiles: Standard Methods Comparison. *Ann. Microbiol.* **2011**, *61*, 493–498, doi:https://doi.org/10.1007/s13213-010-0163-8.
3. Ceballos, R.L.; von Bilderling, C.; Guz, L.; Bernal, C.; Famá, L. Effect of Greenly Synthetized Silver Nanoparticles on the Properties of Active Starch Films Obtained by Extrusion and Compression Molding. *Carbohydr. Polym.* **2021**, *261*, 117871, doi:https://doi.org/10.1016/j.carbpol.2021.117871.
4. He, Q.; Zhang, Y.; Cai, X.; Wang, S. Fabrication of Gelatin–TiO2 Nanocomposite Film and Its Structural, Antibacterial and Physical Properties. *Int. J. Biol. Macromol.* **2016**, *84*, 153–160.
5. Tang, Z.; Chen, C.; Xie, J. Development of Antimicrobial Active Films Based on Poly (Vinyl Alcohol) Containing Nano-TiO2 and Its Application in Macrobrachium Rosenbergii Packaging. *J. Food Process. Preserv.* **2018**, *42*, e13702, doi:https://doi.org/10.1111/jfpp.13702.
6. Sethy, N.K.; Arif, Z.; Mishra, P.K.; Kumar, P. Nanocomposite Film with Green Synthesized TiO2 Nanoparticles and Hydrophobic Polydimethylsiloxane Polymer: Synthesis, Characterization, and Antibacterial Test. *J. Polym. Eng.* **2020**, *40*, 211–220, doi:https://doi.org/10.1515/polyeng-2019-0257.
7. Xing, Y.; Li, X.; Zhang, L.; Xu, Q.; Che, Z.; Li, W.; Bai, Y.; Li, K. Effect of TiO2 Nanoparticles on the Antibacterial and Physical Properties of Polyethylene-Based Film. *Prog. Org. Coat.* **2012**, *73*, 219–224, doi:https://doi.org/10.1016/j.porgcoat.2011.11.005.
