# Supplementary figures and images for: Effect of TiO2 Nanoparticles and Extrusion Process on the Physicochemical Properties of Biodegradable and Active Cassava Starch Nanocomposites
Source: Polymers (Basel). 2023 Jan 20;15(3):535. doi: 10.3390/polym15030535 (PMC9918894; doi:10.3390/polym15030535)

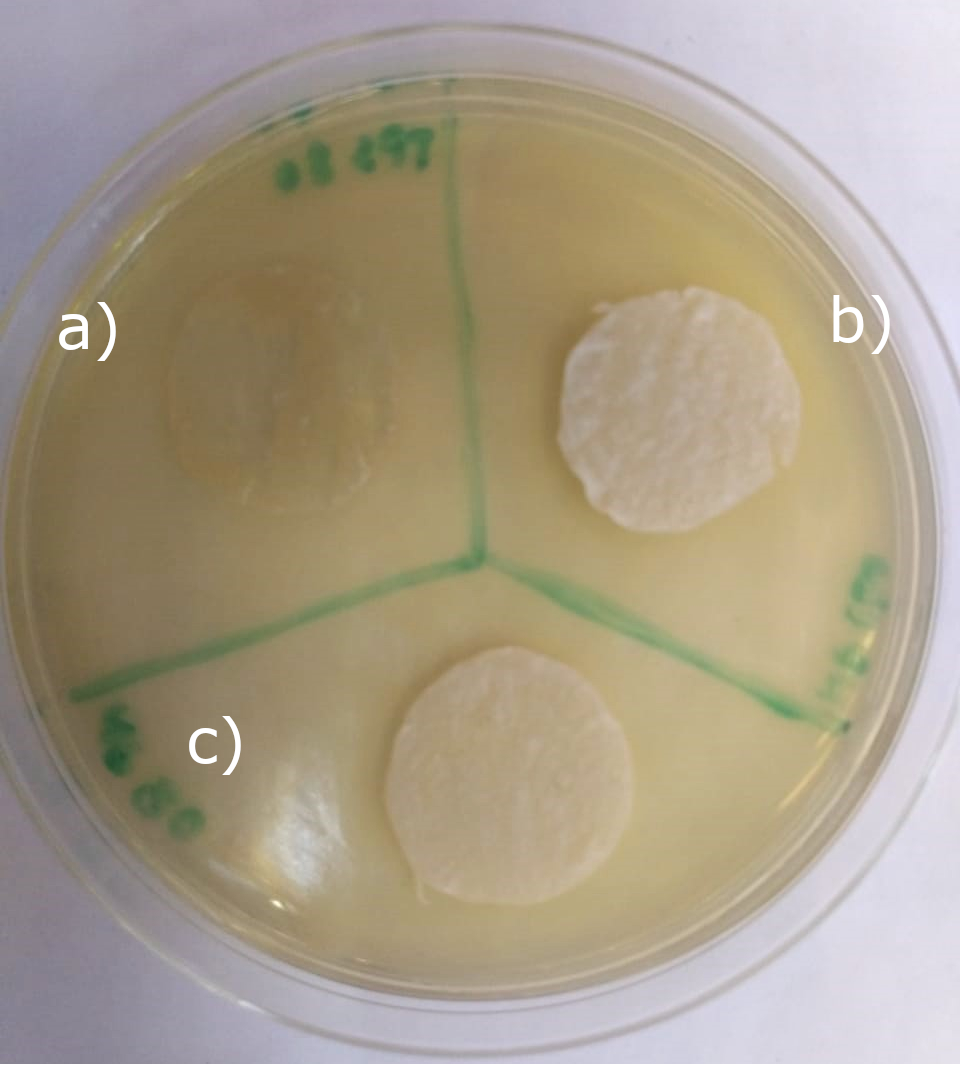

Supplement: Supplementary file 1 [file polymers-15-00535-s001.zip › polymers-2074753-supplementary/Figure S1.jpg]
